# Molecular subtypes of breast cancer predicting clinical benefits of radiotherapy after breast-conserving surgery: a propensity-score-matched cohort study

**DOI:** 10.1186/s13058-023-01747-9

**Published:** 2023-12-08

**Authors:** Shih-Kai Hung, Hsuan-Ju Yang, Moon-Sing Lee, Dai-Wei Liu, Liang-Cheng Chen, Chia-Hui Chew, Chun-Hung Lin, Cheng-Hung Lee, Szu-Chin Li, Chung-Lin Hong, Chih-Chia Yu, Ben-Hui Yu, Feng-Chun Hsu, Wen-Yen Chiou, Hon-Yi Lin

**Affiliations:** 1 Department of Radiation Oncology, Dalin Tzu Chi Hospital, Buddhist Tzu Chi Medical Foundation, Chiayi, Taiwan; 2 School of Medicine, Tzu Chi University, Hualien, Taiwan; 3 Departments of Radiation Oncology, Hualien Tzu Chi Hospital, Buddhist Tzu Chi Medical Foundation, Hualien, Taiwan; 4 Department of Computer Science and Information Engineering, National Cheng Kung University, Tainan, Taiwan; 5 Department of General Surgery, Dalin Tzu Chi Hospital, Buddhist Tzu Chi Medical Foundation, Chiayi, Taiwan; 6 Division of Hematology-Oncology, Department of Internal Medicine, Dalin Tzu Chi Hospital, Buddhist Tzu Chi Medical Foundation, Chiayi, Taiwan; 7 Department of Medical Research, Dalin Tzu Chi Hospital, Buddhist Tzu Chi Medical Foundation, Chiayi, Taiwan; 8 Department of Biomedical Sciences, National Chung Cheng University, Min-Hsiung, Chiayi, Taiwan

**Keywords:** Molecular subtypes, Breast-conserving surgery (BCS), Radiotherapy (RT), Propensity-score match (PSM), Breast cancer

## Abstract

**Background:**

Based on the molecular expression of cancer cells, molecular subtypes of breast cancer have been applied to classify patients for predicting clinical outcomes and prognosis. However, further evidence is needed regarding the influence of molecular subtypes on the efficacy of radiotherapy (RT) after breast-conserving surgery (BCS), particularly in a population-based context. Hence, the present study employed a propensity-score-matched cohort design to investigate the potential role of molecular subtypes in stratifying patient outcomes for post-BCS RT and to identify the specific clinical benefits that may emerge.

**Methods:**

From 2006 to 2019, the present study included 59,502 breast cancer patients who underwent BCS from the Taiwan National Health Insurance Research Database. Propensity scores were utilized to match confounding variables between patients with and without RT within each subtype of breast cancer, namely luminal A, luminal B/HER2-negative, luminal B/HER2-positive, basal-like, and HER2-enriched ones. Several clinical outcomes were assessed, in terms of local recurrence (LR), regional recurrence (RR), distant metastasis (DM), disease-free survival (DFS), and overall survival (OS).

**Results:**

After post-BCS RT, patients with luminal A and luminal B/HER2-positive breast cancers exhibited a decrease in LR (adjusted hazard ratio [aHR] = 0.18, *p* < 0.0001; and, 0.24, *p* = 0.0049, respectively). Furthermore, reduced RR and improved DFS were observed in patients with luminal A (aHR = 0.15, *p* = 0.0004; and 0.29, *p* < 0.0001), luminal B/HER2-negative (aHR = 0.06, *p* = 0.0093; and, 0.46, *p* = 0.028), and luminal B/HER2-positive (aHR = 0.14, *p* = 0.01; and, 0.38, *p* < 0.0001) breast cancers. Notably, OS benefits were found in patients with luminal A (aHR = 0.62, *p* = 0.002), luminal B/HER2-negative (aHR = 0.30, *p* < 0.0001), basal-like (aHR = 0.40, *p* < 0.0001), and HER2-enriched (aHR = 0.50, *p* = 0.03), but not luminal B/HER2-positive diseases. Remarkably, when considering DM, luminal A patients who received RT demonstrated a lower cumulative incidence of DM than those without RT (*p* = 0.02).

**Conclusion:**

In patients with luminal A breast cancer who undergo BCS, RT could decrease the likelihood of tumor metastasis. After RT, the tumor’s hormone receptor status may predict tumor control regarding LR, RR, and DFS. Besides, the HER2 status of luminal breast cancer patients may serve as an additional predictor of OS after post-BCS RT. However, further prospective studies are required to validate these findings.

## Introduction

Breast-conserving therapy, comprising breast-conserving surgery (BCS) followed by radiotherapy (RT), is a standard approach in treating patients with early-stage breast cancer [1]. Post-BCS RT has been proven effective in preventing tumor recurrence by promoting cell apoptosis and inhibiting cell cycle progression [2–4]. Numerous studies have shown improved clinical outcomes in post-BCS RT patients [5–7]. However, the benefits of RT may vary among postoperative patients, possibly due to the biological heterogeneity of breast tumors [8].

The diverse nature of breast tumors at the molecular level leads to variations in the presentation of breast cancer. Through the use of immunohistochemistry (IHC) staining and fluorescence in situ hybridization (FISH) analyses, the main molecular subtypes of breast cancer were identified, as follows: luminal A (Hormone receptor-positive/Human Epidermal Growth factor Receptor-2 negative; HR+/HER2−), luminal B (HR+/HER2+and HR+/HER2−), basal-like (HR−/HER2−), and HER2-enriched (HR−/HER2 +) [9]. Different subtypes of breast cancer have been found to have varying prognoses depending on the treatments applied [8, 10, 11]. However, previous studies have presented contradictory and incomplete findings regarding the role of molecular subtypes in the outcomes of postoperative RT. For example, while one study showed a slight benefit of RT in luminal A breast cancer patients [12], another study did not find such benefits [13]. Furthermore, there is limited information on the benefits of post-BCS RT for patients with different molecular subtypes, particular from population-based evidence. It is important to note that previous studies have had limitations in their research methodology, as they failed to account for potential confounding factors such as the surgical approach [14, 15] and risk factors associated with specific breast cancer subtypes [16–18]. These factors have been shown to impact clinical outcomes [19, 20] and should be considered for future research.

In the present study, we employed the Taiwan National Health Insurance Research Database (TNHIRD) to examine the effects of different breast cancer subtypes on clinical outcomes in patients who underwent post-BCS RT. We conducted a propensity-score-matched cohort design to investigate this topic comprehensively. The main goal was to explore whether there were notable variations in the clinical benefits of post-BCS RT across various molecular subtypes of breast cancer.

## Materials and methods

### Database

The present study analyzed clinical information from the TNHIRD. This database is extensive and includes comprehensive clinical information for over 99% of the population in Taiwan. The database undergoes strict and regular evaluation by the National Health Insurance Administration (NHIA) [19].

### Study population

The present study included female patients diagnosed with breast cancer from 2006 to 2019. The patients were selected based on the International Classification of Disease coding criteria, ninth revision, Clinical Modification (ICD-9-CM), with a breast cancer diagnosis (ICD-9-CM code 174.0–174.9). To ensure the accuracy of the data, we combined the breast cancer diagnostic code from ICD-9-CM with the registry code for patients with catastrophic illnesses. This approach allowed us to confirm that the selected data corresponded to actual records of breast cancer cases.

**Fig. 1:**
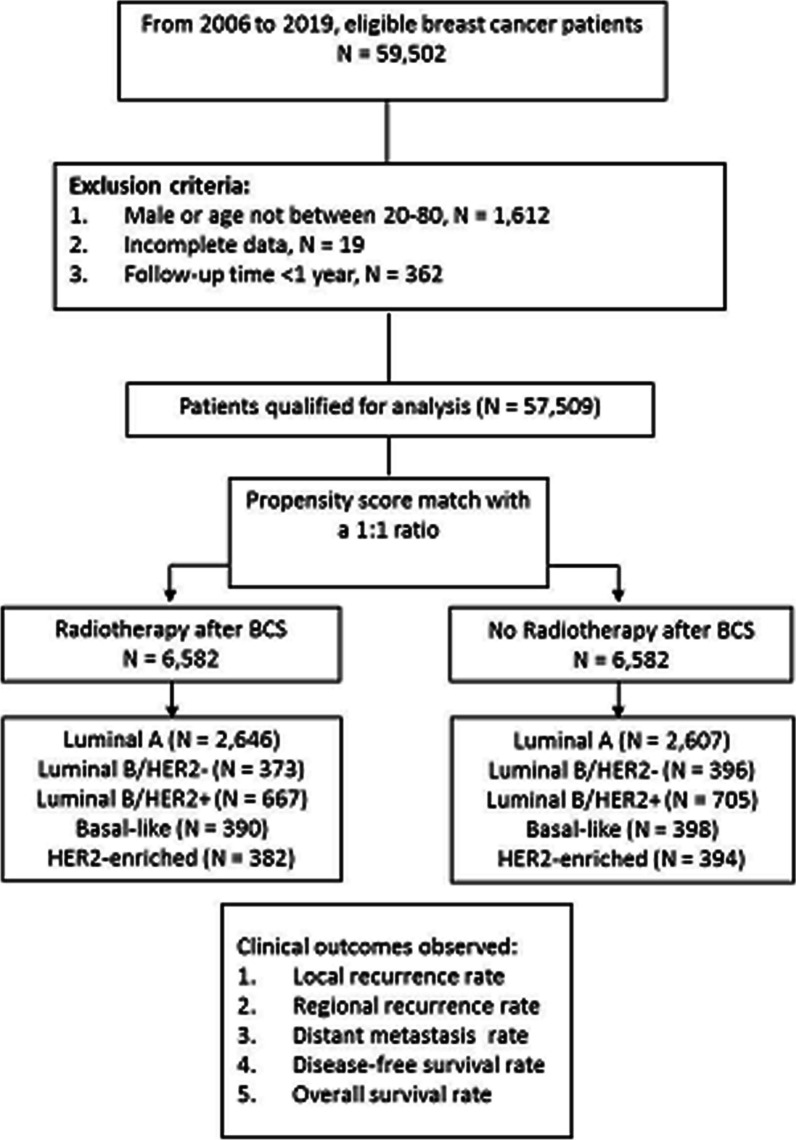
Patient allocation flow chart

Inclusion criteria were as follows: adult patients aged 20–80 who underwent BCS, had clinical stages ranging from stage 0 to stage IV with a follow-up period of more than one year after breast cancer treatment. To compare the outcomes, eligible patients who underwent BCS were divided into two groups using propensity score matching: those who received RT and those who did not. Furthermore, we categorized the patients into five molecular breast cancer subtypes within each group, as shown in Fig. 1.

Table 1 shows the criteria of breast cancer subtypes, according to the St. Gallen International Expert Consensus [20], as follows: luminal A, luminal B/HER2 negative, luminal B/HER2-positive, basal-like, and HER2-enriched. Because information on Ki-67 was unavailable in the TNHIRD, the histological grade was used as an alternative measure of cell proliferation, as recommended in previous studies [21, 22].

**Table 1:** The classification of five molecular subtypes of breast cancer

Subtypes	ER	PR	HER 2	Grade
	+	+		
Luminal A	+	−	−	1, 2
	−	+		
	+	+	−	3
Luminal B/HER2 negative	+	−		
	−	+		
	+	+	+	All
Luminal B/HER2 positive	+	−		
	−	+		
Basal-like	−	−	−	All
HER2-enriched	−	−	+	All

### Endpoints

Five clinical outcomes were measured as study endpoints, as follows: local recurrence (LR), regional recurrence (RR), distant metastasis (DM), disease-free survival (DFS), and overall survival (OS). These clinical outcomes were determined based on pathology information or CT/MRI images. The endpoint day was set as the day when the first event occurred. The follow-up time was calculated from the day of primary breast cancer diagnosis to either the day of most recent visit or the event of occurrence.

### Statistical analysis

The RT patients were matched in a 1:1 ratio with non-RT patients based on several confounding factors, including age, molecular subtypes, clinical and pathological stage, comorbidities, chemotherapy, hormone therapy, target therapy, anti-cancer agents, and socioeconomic status. The patient characteristics and clinical information are summarized in Table 2. The RT and non-RT cohorts were compared within each molecular subtype. We utilized the Fine and Gray's competing-risk regression model to estimate hazard ratios (HRs) and 95% confidence intervals (CIs) for the time-to-event clinical outcomes. All clinical outcomes were analyzed using multivariate analysis to adjust confounding factors. We used the SAS software to conduct the statistical analysis (version 9.2; SAS, Inc., Cary, NC). A two-sided test with a P-value of less than 0.05 was considered statistically significant.

**Table 2:** Information of post-BCS patients’ characteristics

Variables	Post-BCS patients after 1:1 PS match			*p* value
	RT *N* = 6582 (%)		No RT *N* = 6582 (%)	
Age group (years)				0.96
20–46	1578 (23.97)		1594 (24.22)	
47–52	1490 (22.64)		1468 (22.30)	
53–60	1534 (23.31)		1542 (23.43)	
> 60	1980 (30.08)		1978 (30.05)	
Subtype				0.74
Luminal A	2646 (40.20)		2607 (39.61)	
Luminal B/HER2 -	373 (5.67)		396 (6.02)	
Luminal B/HER2+	667 (10.13)		705 (10.71)	
Basal-like	390 (5.93)		398 (6.05)	
HER2-enriched	382.00 (5.80)		394 (5.99)	
Unknown	2124 (32.27)		2082 (31.63)	
RT dose (cGy)				< 0.0001
0	–		6582 (100)	
3000–5040	711 (10.80)		–	
5040–6040	4037 (61.33)		–	
> 6040	1834 (27.86)		–	
C-stage				0.42
0	423 (6.43)		445 (6.76)	
I	3231 (49.09)		3173 (48.21)	
II	2607 (39.61)		2627 (39.91)	
III	208 (3.61)		237 (3.60)	
IV	113 (1.72)		100 (1.52)	
P-stage				0.21
0	184 (2.80)		194 (2.95)	
I	3412 (51.84)		3307 (50.24)	
II	2300 (34.94)		2330 (35.40)	
III	310 (4.71)		364 (5.53)	
IV	108 (1.64)		102 (1.55)	
Unknown	268 (4.07)		285 (4.33)	
Comorbidities				
COPD	263 (4.00)		292 (4.44)	0.20
Hypertension	1565 (23.78)		1537 (23.35)	0.56
Diabetes mellitus	667 (10.13)		710 (10.79)	0.22
CKD	132 (2.01)		153 (2.32)	0.20
Heart failure	40 (0.61)		46 (0.70)	0.51
Liver disease	326 (4.95)		371 (5.64)	0.07
Liver cirrhosis	24 (0.36)		23 (0.35)	0.88
Chemotherapy	3083 (46.84)		3101 (47.11)	0.75
Hormone therapy	5016 (76.21)		4959 (75.34)	0.24
Target therapy	345 (5.24)		400 (6.08)	0.03
Anti-cancer agents				
Doxorubicin	616 (9.36)		464 (7.05)	< 0.0001
Epirubicin	1797 (27.30)		1709 (25.96)	0.08
Docetaxel	1297 (19.71)		1037 (15.76)	< 0.001
Paclitaxel	189 (2.87)		206 (3.13)	0.38
Carboplatin	15 (0.23)		22 (0.33)	0.24
Cyclophosphamide	2895 (43.98)		2827 (42.95)	0.23
Fluorouracil	1795 (27.27)		1804 (27.41)	0.86
Methotrexate	218 (3.31)		283 (4.30)	0.003
Family income (NTD per month)				0.95
< 20,100	1636 (24.86)		1653 (25.11)	
20,101–22,800	1138 (17.29)		1148 (17.44)	
22,801–42000	2007 (30.49)		1978 (30.05)	
> 42,000	1801 (27.36)		1803 (27.39)	
Urbanization level				0.70
City	1817 (27.61)		1776 (26.98)	
Satellite cities	3462 (52.60)		3482 (52.90)	
Rural areas	1303 (19.80)		1324 (20.12)	
Geographic region				0.06
North	3016 (45.82)		3064 (46.55)	
Central	1637 (24.87)		1630 (24.76)	
South	1839 (27.94)		1764 (26.80)	
East	90 (1.37)		124 (1.88)	

*PS* propensity score, *BCS* breast-conserving surgery, *RT* radiotherapy, *C-stage* clinical stage, *P-stage* pathological stage, *BCS* breast-conserving surgery, *COPD* chronic obstructive pulmonary disease, *CKD* chronic kidney disease, *NTD* New Taiwan dollar, Insurance premium for National Health Insurance is according to family income

## Results

From 2006 to 2019, 59,502 breast cancer patients who underwent BCS were included. After applying the exclusion criteria (Fig. 1), 57,509 patients met the requirements for further analysis. Through a 1:1 matching, we obtained two cohorts of post-BCS patients: the RT (*n* = 6582) and non-RT ones (*n* = 6582; Table 2). Among the specific molecular subtype groups, the numbers of patients with and without RT were as follows: luminal A (*n* = 2646 and 2607), luminal B/HER2-negative (*n* = 373 and 396), luminal B/HER2-positive (*n* = 667 and 705), basal-like (*n* = 390 and 398), and HER2-enriched breast cancer (*n* = 382 and 394), respectively.

RT improved LR (aHR, 0.33; 95% CI 0.25–0.44; *p* < 0.0001), RR (aHR, 0.29; 95% CI 0.19–0.43; *p* < 0.0001), DM (aHR, 0.81; 95% CI 0.66–0.99; *p* < 0.05), DFS (aHR, 0.50; 95% CI 0.43–0.58; *p* < 0.0001), and OS (aHR, 0.51; 95% CI 0.45–0.58; *p* < 0.0001).

RT provided the most advantage in reducing rates of LR (66%) and RR (72%), surpassing a 50% reduction. Subsequently, it exhibited reduction rates of 48% in DFS and 46% in OS. Although the reduction rate of DM (19%) was the lowest, post-BCS patients still experienced statistically significant benefits from RT (aHR = 0.81, *p* < 0.05; Table 3).

**Table 3:** Overall prognosis after radiotherapy in post-BCS patients

	Total (N)	Event (N)	Incidence rate (%)	Reduction rate (%)	aHR	95% CI	*p* value
LR				66	0.33	0.25–0.44	< 0.0001
No RT	6472	202	3.1				
RT	6565	69	1.1				
Total	13,164	271	2.1				
RR				72	0.29	0.19–0.43	< 0.0001
No RT	6538	105	1.6				
RT	6574	30	0.5				
Total	13,112	135	1.0				
DM				19	0.81	0.66–0.99	0.044
No RT	6520	218	3.3				
RT	6561	177	2.7				
Total	13,081	395	3.0				
DFS				48	0.50	0.43–0.58	< 0.0001
No RT	6375	509	8.0				
RT	6537	270	4.1				
Total	12,912	779	6.0				
OS				46	0.51	0.45–0.58	< 0.0001
No RT	6582	715	10.9				
RT	6582	387	5.9				
Total	13,164	1102	8.4				

*RT* radiotherapy, *LR* local recurrence, *RR* regional recurrence, *DM* distant metastasis, *DFS* disease-free survival, *OS* overall survival; *aHR* adjusted hazard ratio, *95% CI* 95% confidence interval

aHR was obtained from multivariate analysis which was based on Fine and Gray competing risks proportional hazards regression model by adjusting age, molecular subtypes of breast tumor, clinical and pathological stage, comorbidities, chemotherapy, hormone therapy, target therapy, anti-cancer agents, and socioeconomic status

Table 4 presents the variations in prognosis after RT among five molecular subtypes of breast cancer. After adjusting confounding factors, we observed RT improved LR in luminal A (aHR = 0.18, *p* < 0.0001) and luminal B/HER2-positive breast cancers (aHR = 0.24, *p* = 0.0049). Furthermore, RT improved RR and DFS in luminal A (aHR = 0.15, *p* = 0.0004 and 0.29, *p* < 0.0001), luminal B/HER2-negative (aHR = 0.06, *p* = 0.0093 and 0.46, *p* = 0.028), and luminal B/HER2-positive breast cancers (aHR = 0.14, *p* = 0.01 and 0.38, *p* < 0.0001), respectively.

**Table 4:** Adjusted hazard ratio of clinical outcomes after radiotherapy in various subtypes of breast cancer

Subtype	LR		RR		DM		DFS		OS	
	aHR	95% CI	aHR	95% CI	aHR	95% CI	aHR	95% CI	aHR	95% CI
Luminal A	0.18***	0.09–0.34	0.15**	0.05–0.42	0.59	0.34–1.01	0.29***	0.20–0.42	0.62**	0.45–0.85
Luminal B/HER2-	0.22	0.03–1.80	0.06**	0.01–0.50	0.89	0.34–2.30	0.46*	0.23–0.92	0.30***	0.17–0.54
Luminal B/HER2+	0.24**	0.09–0.65	0.14*	0.03–0.63	0.68	0.36–1.27	0.38***	0.24–0.62	0.71	0.45–1.11
Basal-like	0.21	0.03–1.61	0.23	0.04–1.37	1.13	0.45–2.81	0.65	0.32–1.28	0.40***	0.25–0.63
HER2-enriched	0.79	0.30–2.14	0.90	0.12–6.50	0.74	0.29–1.92	0.85	0.47–1.54	0.50*	0.26–0.97

*LR* local recurrence; *RR* regional recurrence; *DM* distant metastasis; *DFS* disease-free survival; *OS* overall survival; *aHR* adjusted hazard ratio

aHR was obtained from multivariate analysis based on Fine and Gray competing risks proportional hazards regression model by adjusting age, clinical and pathological stage, comorbidities, chemotherapy, hormone therapy, target therapy, anti-cancer agents, and socioeconomic status

*p < 0.05; **p < 0.01; ***p < 0.0001

The analysis also revealed OS benefits in both luminal and non-luminal breast cancers, including luminal A (aHR = 0.62, *p* = 0.002), luminal B/HER2-negative (aHR = 0.30, *p* < 0.0001), Basal-like (aHR = 0.40, *p* < 0.0001), and HER2-enriched (aHR = 0.50, *p* = 0.03), except for luminal B/HER2-positive breast cancers. Besides, a lower cumulative incidence of DM was observed only in the group of luminal A breast cancer patients with RT than those without RT (*p* = 0.02).

**Fig. 2:**
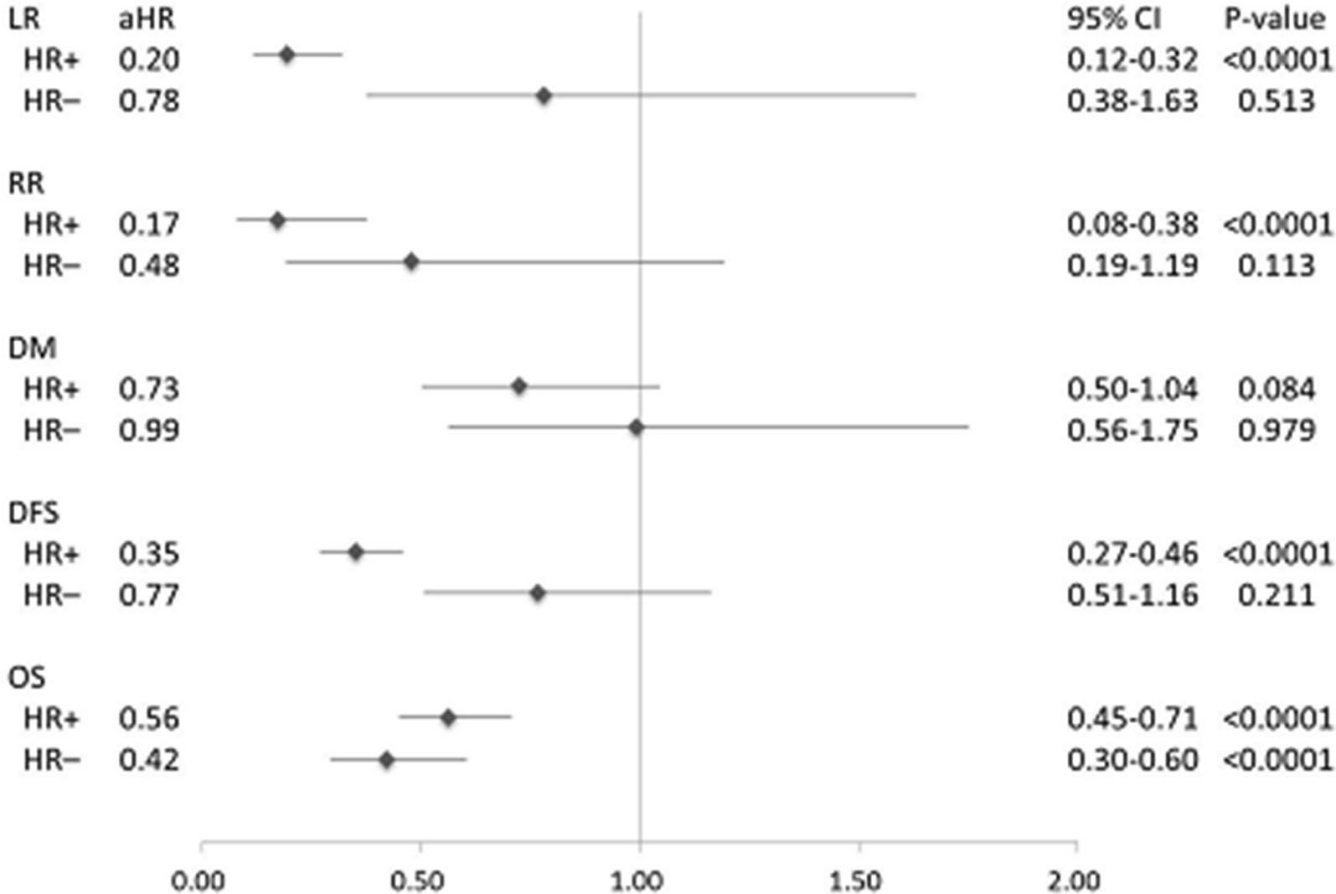
A forest plot for clinical outcomes after radiotherapy in post-BCS patients with HR+and HR− breast cancer

The luminal breast cancer (hormone receptor-positive; HR+) generally showed positive outcomes in terms of LR, RR, and DFS after post-BCS RT (aHR = 0.20, 0.17, and 0.35, respectively). However, non-luminal breast cancer (hormone receptor-negative; HR−) did not exhibit the same benefits (aHR = 0.78, 0.48, and 0.77, respectively; Fig. 2). We also observed that the HR+and HR− groups benefited from post-BCS RT regarding OS (aHR = 0.56 and 0.42). However, the risk of DM did not noticeably decrease after RT in either group (aHR = 0.73 and 0.99, respectively).

**Fig. 3:**
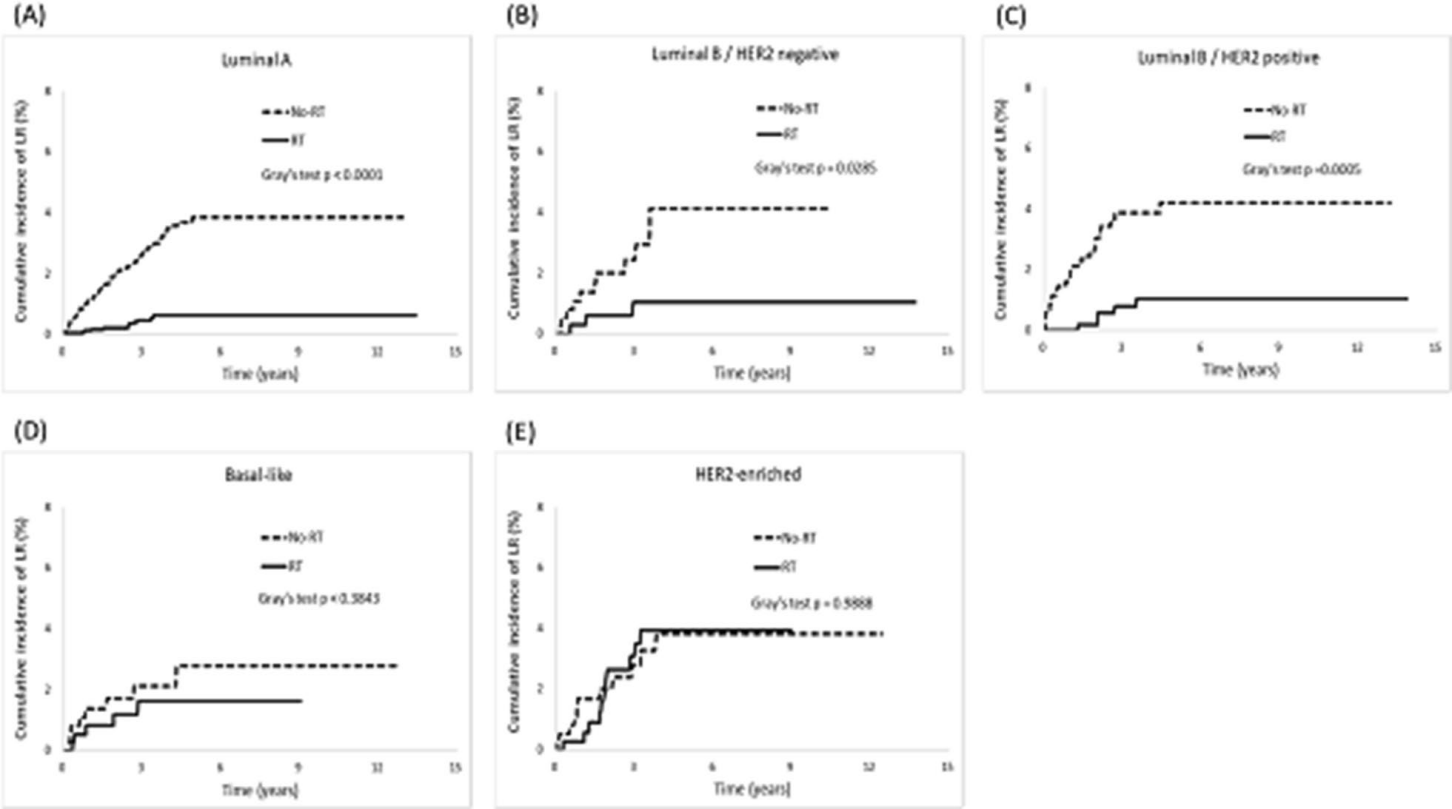
Cumulative incidence of local recurrence (LR)

**Fig. 4:**
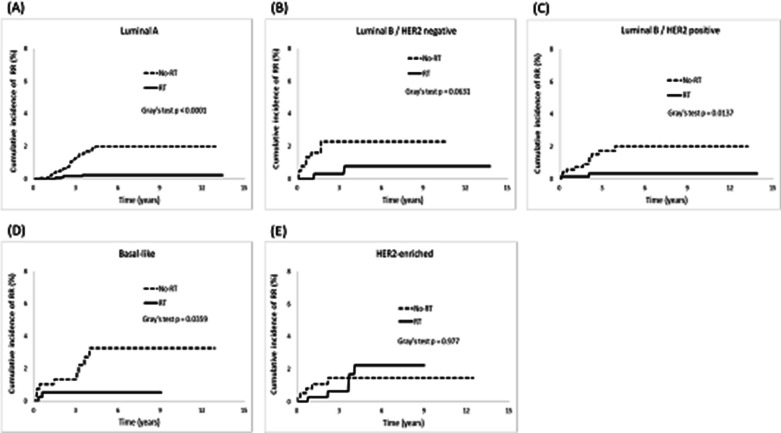
Cumulative incidence of regional recurrence (RR)

Furthermore, we observed RT patients a lower cumulative incidence of LR than non-RT patients in the luminal A (*p* < 0.0001), luminal B/HER2-negative (*p* = 0.02), and luminal B/HER2-positive breast cancer groups (*p* = 0.005; Fig. 3). Subsequently, a resulting increased DFS was observed, with higher rates in the luminal A (*p* < 0.0001), luminal B/HER2-negative (*p* = 0.01), and luminal B/HER2-positive breast cancer groups (*p* < 0.0001; Fig. 6). Regarding the cumulative incidence of RR (Fig. 4), patients benefited from RT were in the luminal A (*p* < 0.0001) and luminal B/HER2-positive breast cancer groups (*p* = 0.01). Considering OS (Fig. 7), patients benefited from RT were in the luminal A (*p* < 0.0001), luminal B/HER2-negative breast cancer (*p* < 0.0001), Basal-like (*p* = 0.0003), and HER2-enriched breast cancer groups (*p* = 0.02).

**Fig. 5:**
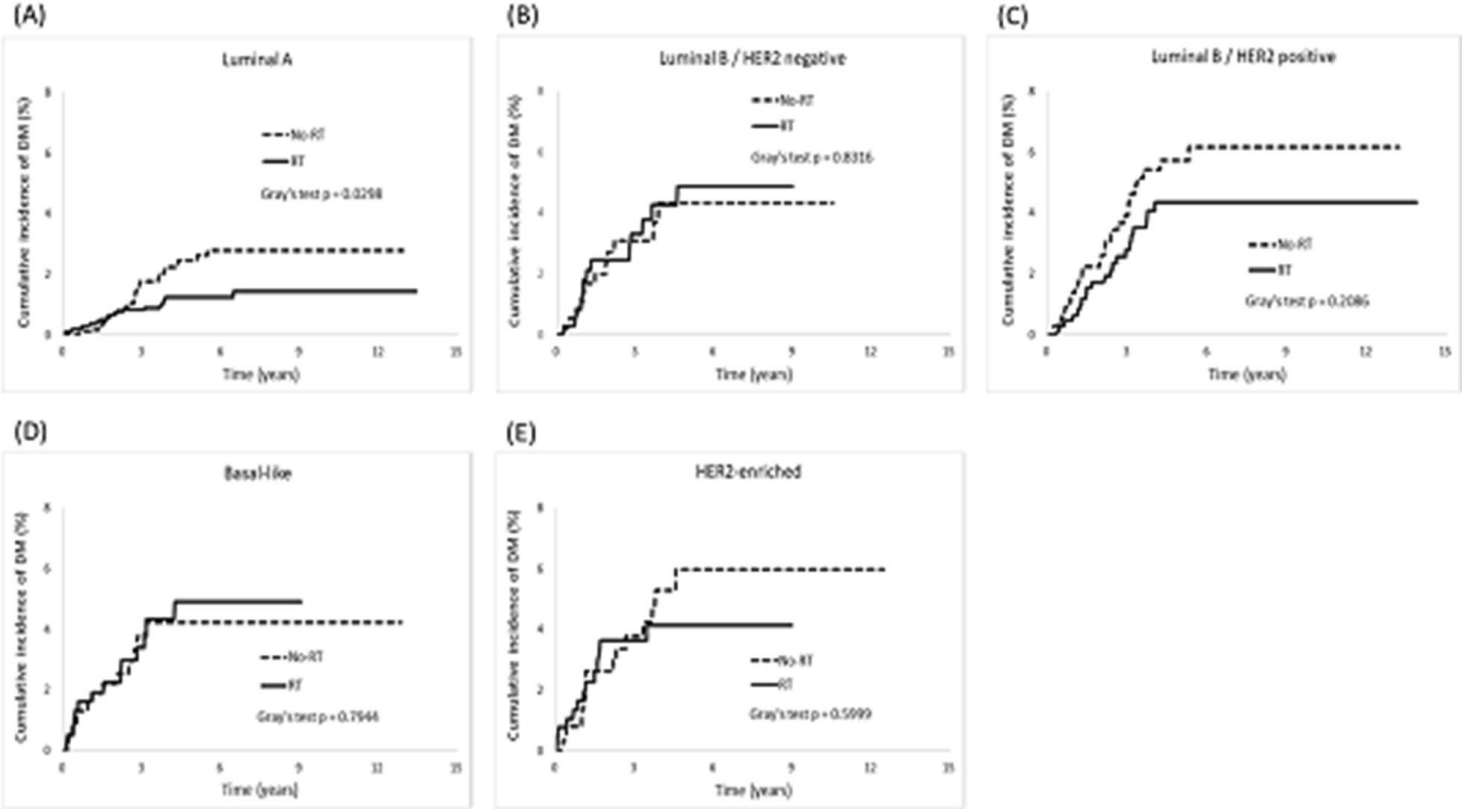
Cumulative incidence of distant metastasis (DM)

When compared with non-RT patients, a lower cumulative incidence of DM was only observed in RT patients in the luminal A breast cancer group (*p* = 0.02; Fig. 5). We further examined the incidences of DM between the RT and non-RT cohorts for each molecular subtype group among post-BCS patients (Table 5).

**Table 5:** The comparison of distant metastasis incidences between post-BCS patients with and without radiotherapy in each group of molecular subtypes

Subtype	Post-BCS patients after PS match				*p* value
	RT		No RT		
	N	(%)	N	(%)	
Luminal A	24	0.91	39	1.50	0.048*
Luminal B/HER2−	13	3.51	12	3.06	0.726
Luminal B/HER2+	21	3.17	30	4.33	0.261
Basal-like	14	3.61	13	3.32	0.828
HER2-enriched	13	3.41	16	4.13	0.599
HR +	58	1.58	81	2.20	0.049*
HR−	27	3.51	29	3.73	0.819

*BCS* breast-conserving surgery; *PS* propensity score; *HR* hormone receptor; *RT* radiotherapy

Note that patients in RT and No RT cohorts were matched by age, clinical and pathological stage, comorbidities, chemotherapy, hormone therapy, target therapy, anti-cancer agents, and socioeconomic status

*p < 0.05

## Discussion

**Fig. 6:**
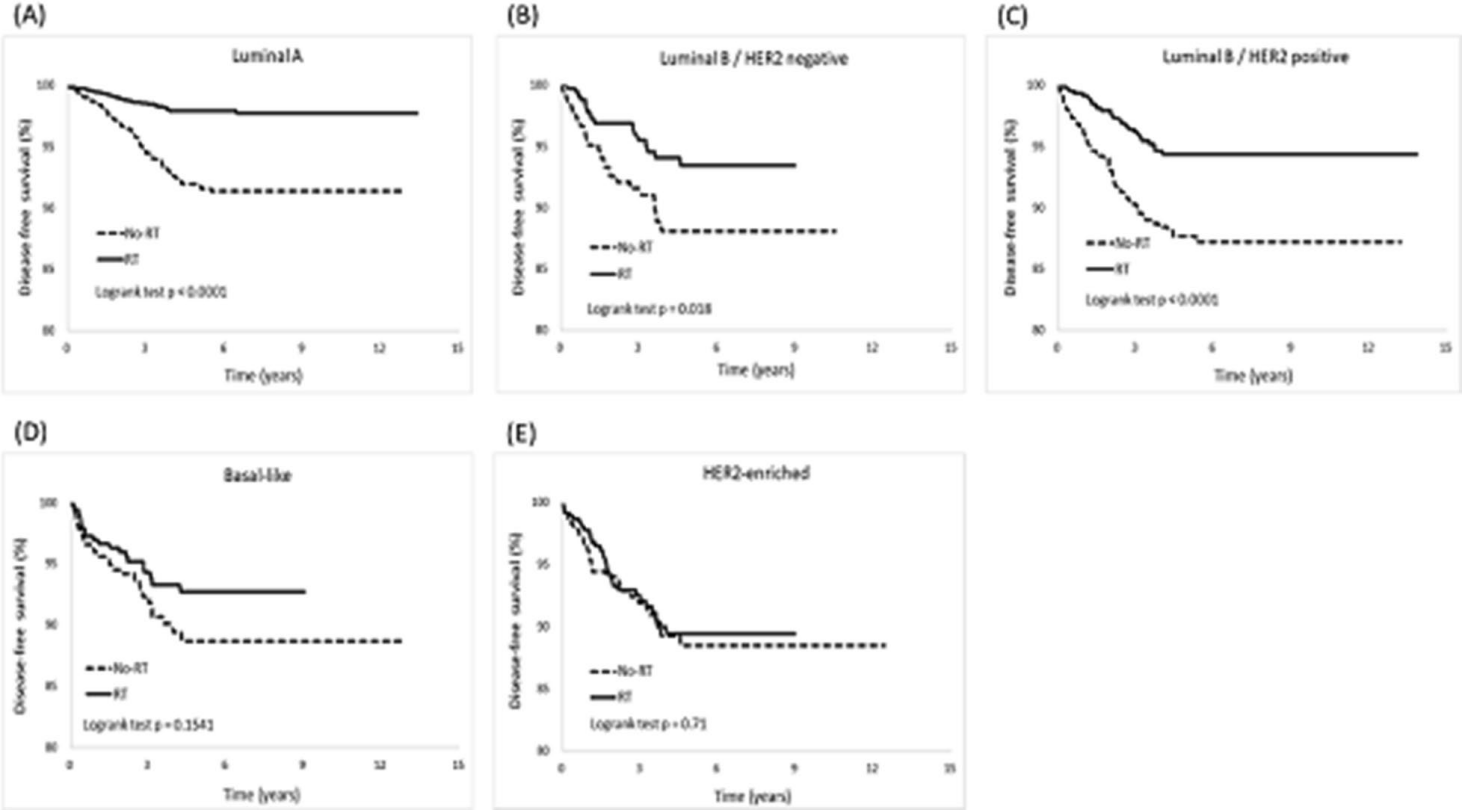
Cumulative incidence of disease-free survival (DFS)

**Fig. 7:**
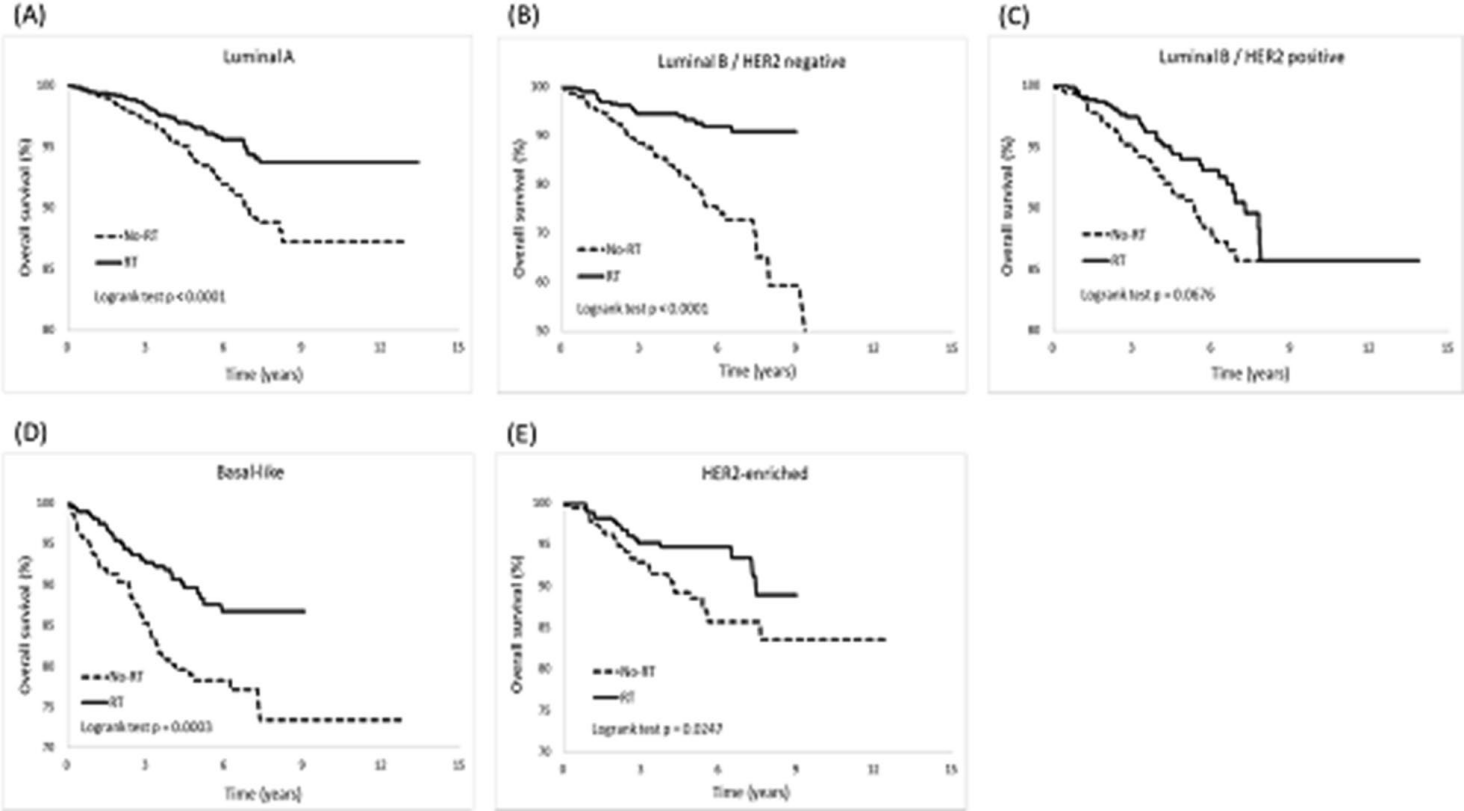
Cumulative incidence of overall survival (OS)

The present study investigated how different molecular subtypes of breast cancer affect clinical outcomes after post-BCS RT. In general, post-BCS RT reduced the risks of LR, RR, and DM and prolonged DFS and OS rates in breast cancer patients. However, we discovered that not all molecular subtypes derived equal benefits from RT, and the advantages of RT were not consistent across all clinical outcomes. Our findings suggest that the intrinsic molecular subtypes of breast cancer can influence the clinical outcomes following RT in different ways, as follows. First, RT patients with luminal breast cancer experienced significant benefits, including lower risks of LR and RR and prolonged DFS. Conversely, patients with non-luminal breast cancer showed limited advantages in these clinical outcomes (Table 4 and Figs. 2, 3, 4, and 6). Second, whether the breast cancer was classified as luminal or non-luminal, post-BCS patients benefited from RT regarding extended OS. However, patients with HR+/HER2+breast cancer did not experience the same benefits (Table 4, Figs. 2 and 7). Third, regarding the observation of DM risk, RT was beneficial in reducing the probability of tumor metastasis after BCS only for patients with luminal A breast cancer (Table 4, Figs. 2 and 5).

In the era of precision medicine, the application of molecular classification of breast cancer is developing actively. The influence of diverse breast cancer subtypes on prognosis and treatment response is highly valued [23, 24]. When considering RT for postoperative breast cancer patients, clinical factors such as tumor stage, lymph node invasion, and surgical margin play a crucial role. However, advancements in molecular biotechnology have revealed the heterogeneity within breast tumors, leading to the recognition of the clinical significance of classifying breast cancer into intrinsic subtypes [25]. Our study suggested that molecular subtypes have a predictive role in determining the clinical benefits of post-BCS RT. This information can be valuable for clinicians in developing personalized cancer therapies.

Breast tumors vary in their response to treatment [26], and the effectiveness of RT in improving clinical outcomes may depend on the different gene expression patterns of these tumors [21]. When compared with hormone receptor-negative (HR−) tumors, our findings revealed that RT improved LR, RR, and DFS in hormone receptor-positive (HR+) tumors. This finding suggests that the hormone receptor status can be a predictive marker for tumor control following RT. [27]

Studies have shown that luminal breast cancer generally has more favorable outcomes after RT compared to non-luminal breast cancer [8, 21, 28, 29], and distinct biological processes have been identified that impact prognosis and treatment response in estrogen receptor-positive (ER-positive) and estrogen receptor-negative (ER-negative) breast cancers [30]. Furthermore, previous research has proposed different levels of sensitivity to radiation in individual breast cancer subtypes, suggesting that the differences in prognosis between luminal and non-luminal breast cancers may be attributed to significantly enhanced radiosensitivity in luminal breast cancer. In contrast, HER2-enriched [8] and basal-like breast cancers exhibit strong resistance to radiation [31]. The estrogen receptor has been found to interact with the androgen receptor, further enhancing the radiosensitivity of breast tumors [32]. Inhibiting estrogen receptor signaling has also been shown to increase the sensitivity of ER-positive tumors to radiation [33]. Additionally, clinical studies have pointed out the association between poor prognosis and radioresistance in HER2-overexpressing and triple-negative breast tumors [27, 34].

Emerging evidence has shed light on potential mechanisms contributing to the robust radioresistance observed in HER2-enriched breast cancer. These mechanisms include the HER2-NF-kappaB-HER2 loop, which mediates radiation-induced adaptive resistance [34], as well as the Fak-mediated pathway, which enhances radiosensitization in HER2-overexpressing breast cancer [35, 36]. Furthermore, the involvement of microRNA (miRNA) in the regulatory mechanism of triple-negative breast tumors has been discovered. For instance, high expression of MiR27a has been found to play a crucial role in regulating the radiosensitivity of triple-negative breast cancer cells [37, 38].

Notably, while significant tumor control was observed in HR+cancer patients after RT, our analysis revealed that an improved OS was found only in HR+/HER2-, not HR+/HER2+tumors. This finding suggests that HER2 status may further predict the benefits of OS after RT in patients with luminal breast cancer. Even in patients who underwent postmastectomy radiotherapy (PMRT), as studied previously [27], OS benefits from RT were observed solely among those with hormone receptor-positive and HER2-negative breast cancers (including two HR+subtypes) [27]. The resistance of HER2-overexpressing tumors to radiation may affect the OS benefits, even in cases with good prognostic markers for luminal breast cancer (HR +). Moreover, the extent of radioresistance may vary among different cell types of HER2-positive tumors, as previous studies have proposed the existence of inherent radiosensitivity differences in HER2-overexpressing tumor cells, which should be considered in conventional RT approaches [39].

Remarkably, a lower cumulative incidence rate of DM was observed after RT in patients with luminal A breast cancer. This finding is consistent with previous studies [29, 40]. Furthermore, HER2-overexpression has been linked to radioresistance and epithelial-to-mesenchymal transition in breast cancer stem cells [41, 42], which are crucial for tumor invasion, migration, and metastatic outgrowth [43]. Taken together, these characteristics of different breast tumor subtypes may contribute to a high likelihood of metastasis even after RT, but there is still a slightly favorable outcome observed after RT in luminal A (HR+/HER2-) breast tumors.

The present study discovered a significant increase in OS rates among RT patients. This finding indicates that the benefits of OS from post-BCS RT extend beyond just the luminal subtypes and apply to basal-like and HER2-enriched breast cancers. The lack of tumor control in basal-like and HER2-enriched breast cancers did not diminish the OS benefits of post-BCS RT, suggesting that RT has a robust effect on prolonging survival in post-BCS patients [5, 44, 45]. It is a proven fact that post-BCS RT has noticeable positive effects on breast cancer patients, with both breast-cancer-specific survival and OS significantly improved even in elderly patients (age ≥ 70 years) [44]. Furthermore, the benefits of RT on OS were not dependent on irradiation volume, as similar benefits were observed in patients who underwent accelerated partial breast irradiation and those who received whole breast RT [46]. Despite the favorable outcomes associated with post-BCS RT, there remains uncertainty regarding the relationship between molecular subtypes of breast cancer and the benefits of OS from RT due to conflicting previous research [8, 28, 47]. While one study demonstrated a significantly lower 3-year OS rate among patients with the HER2-enriched subtype than those with the luminal subtype [28], another 5-year follow-up study found no differences among molecular subtypes [47].

It should be noted that the influences on the benefits of OS from RT can be multifactorial, including factors such as surgical approach, [14, 15, 48] tumor stage, [49, 50] anti-cancer drug, [51, 52] and age [53], all of which need to be considered when evaluating the impact of molecular subtypes of breast cancer on the clinical benefits of radiotherapy. Therefore, further clinical studies are required to develop a factorial model that incorporates the benefits of overall survival from radiotherapy in molecular subtypes of breast cancer. The present study utilized propensity-score-based matching to control for confounding variables between patients with and without RT, revealing distinct survival benefits from RT in post-BCS patients. Although non-luminal subtypes are recognized as prognostic indicators of breast cancer associated with shorter overall survival [11, 21, 54], the benefits of OS from post-BCS RT should not be disregarded for these subtypes.

The present study has several limitations. Firstly, although the study employed a propensity-score-matched design, it cannot definitively establish a cause-and-effect relationship between breast cancer subtypes and post-RT outcomes due to its retrospective study nature. Secondly, some studies have separately explored the clinical outcomes of RT in pre- [55] and post-menopausal [56] breast cancer patients. However, the correlation between the benefits of RT and menopause status appears to be minimal, as similar clinical outcomes were observed in both groups of patients [57]. Additionally, other factors were not assessed in our study, such as the family history of malignant tumors, lymphovascular invasion [58], physical activity [59, 60], and circulating tumor cell status [61]. These factors could potentially influence the clinical outcomes after RT, despite our efforts to adjust and control for confounding variables.

Remarkably, we should discuss that the present study included BCS patients without RT, which is not the standard care of the current guideline. Among the 57,509 qualified post-BCS patients, we deleted 4648 patients who had no clear tumor stages. In the remaining 52,861 patients, 44,847 RT patients and 8014 non-RT patients were matched with a 1:1 ratio. Therefore, the number of post-BCS patients without RT was accounted for 15.1% of the total number. In the real-world setting, several reasons may result in patients and their families choosing diversely from the standard of care, such as the fear of RT side effects, elderly, economic status, significant comorbidities (e.g., stroke, severe dementia, end-stage renal disease, or heart failure), or other personal factors. Presenting the actual status in a real-world setting is one of the central values of the population-based study. Thus, we co-present the RT and non-RT patients (but match-paired them) instead of excluding the non-RT patients from the present analysis.

## Conclusion

The response of tumors to RT in luminal breast cancer can be predicted by the hormone receptor status, in terms of LR, RR, and DSF. Additionally, HER2 status may serve as an additional predictor for OS following post-BCS RT. The impact of RT in reducing the likelihood of tumor spread after BCS is potentially significant for patients with luminal A breast cancer. However, the lack of tumor control in basal-like and HER2-enriched breast cancers does not affect the OS benefits derived from post-BCS RT.

## Data Availability

Data used in this study are from TNHIRD, and this database is owned by the government. The information contained within the database was authorized for research purposes by the Health and Welfare Data Science Center (HWDC), Ministry of Health and Welfare, Taiwan. The raw data from the TNHIRD is available to the research community; however, the data must be analyzed within the HWDC after the study proposal is approved (https://dep.mohw.gov.tw/dos/np-2497-113.html). The raw data can only be dealt with in this data science center, and cannot be released according to the legal restriction of Data Science Center, Ministry of Health and Welfare, Taiwan. The website of Data Science Center, Ministry of Health and Welfare, Taiwan is as follows: https://dep.mohw.gov.tw/dos/cp-5283-63826-113.html. The confidentiality assurances were addressed by the data regulations of the HWDC. The study protocol, analytic methods, and statistical programming codes are available from the corresponding author on reasonable request.
